# Dysfunction of CD19^+^CD24^hi^CD27^+^ B regulatory cells in patients with bullous pemphigoid

**DOI:** 10.1038/s41598-018-19226-z

**Published:** 2018-01-15

**Authors:** Zhenfeng Liu, Erle Dang, Bing Li, Hongjiang Qiao, Liang Jin, Jieyu Zhang, Gang Wang

**Affiliations:** Department of Dermatology, Xijing Hospital, Fourth Military Medical University, 127 Changlexi Road, Xi’an, 710032 China

## Abstract

Bullous pemphigoid (BP) is an autoimmune blistering skin disease characterized by the production of autoantibodies against the hemidesmosomal protein BP180. B regulatory cells (Bregs) are crucial in maintaining self-tolerance and suppressing autoantibody production. However, it is still unclear whether the dysfunctions of Bregs contributes to the autoantibody production in BP patients. In this study, we found that CD19^+^CD24^hi^CD27^+^ Bregs and IL-10^+^CD19^+^ Bregs were significantly increased in the peripheral blood samples of BP patients compared with that in healthy controls. Moreover, compared to Bregs from healthy individuals, we found that Bregs from BP patients fails to suppress the production of specific anti-BP180 autoantibody when co-cultured with patient-derived PBMCs. Additionally, Bregs from BP patients were defective in suppressing the CD4^+^ T cell proliferation and the cytokines expression (including IFN-γ, TNF-α and IL-4). Notably, we found that patient-derived Bregs produced high level of TNF-α and the TNF inhibitor etanercept could inhibit the autoantibody production in the culture system *in vitro*. Our results indicate that Bregs from BP patient appear phenotypically pro-inflammatory by their cytokine profile and are defective in immunosuppressive function, which suggest that Bregs play a pro-inflammatory role rather than a regulatory role in the pathogenesis of BP.

## Introduction

Bullous pemphigoid (BP) is a prevalent autoimmune blistering disease worldwide, which results from specific antibodies against adhesion molecules BP180 and BP230 of the dermal-epidermal basement membrane zone^1,2^. BP180 is the main pathogenic antigen in BP pathogenesis, with its major epitope of the non-collagenous 16A (NC16A) domain of the juxtamembranous extracellular region^3^. The pathogenic auto-antigen has been identified in BP and the autoantibody production is thought caused by breakdown of self-tolerance^4^. Nevertheless, the mechanism underlying the breakdown of self-tolerance in BP patients is not well-understood.

Regulatory lymphocytes including regulatory T cells (Tregs) and regulatory B cells (Bregs) play crucial roles in maintaining self-tolerance and preventing autoimmunity disorder. These lymphocytes could regulate antibody production by suppressing the activation of T lymphocytes and the co-stimulatory signaling to activate B cells^5^. Recently, several studies showed that the frequency of circulating Tregs was reduced in BP patients, indicating that the dysregulation of Tregs may contribute to the pathogenesis of BP^6^. However, the pathogenic role of the Bregs still need to be explored in BP.

Bregs were identified as a subset of IL-10-producing B cells^7^. Accumulated evidence showed that adoptive transfer of Bregs ameliorates inflammatory response in murine models of autoimmune disease, including Type I diabetes, contact hypersensitivity, and collagen-induced arthritis^8,9^. Recently, Iwata *et al*. confirmed that the CD19^+^IL-10^+^ Bregs can be characterized as CD24^hi^CD27^+^ B cells in humans peripheral blood^10^. These Bregs can suppress the activity of T cells and maintain immune homeostasis mainly via secreting IL-10. In addition, studies showed that in patients with systemic lupus erythematosus (SLE) or diabetes, Bregs showed impairment in immune-suppressive function, thus leading to the breakdown of self-tolerance^11^.

TNF-α is considered to be a critical cytokine for the initiation and perpetuation of inflammation and is mainly produced by activated macrophages and pro-inflammatory lymphocyte^12^. TNF-α contributes to many human autoimmune diseases by promoting the expansion and survival of T cells, including diabetes, rheumatoid arthritis, and psoriasis^13^. It has been reported that β-glucan-activated B lymphocytes could up-regulate pro-inflammatory cytokines TNF-α^14^. And TNF-α and IL-10 play important but opposite role during infection and inflammation^15^. The balance between TNF-α and IL-10 could modulate the effector function of macrophages and cell apoptosis, suggesting an antagonistic effect of TNF-α on immunomodulation of IL-10^16^.

BP is an autoantibody-mediated autoimmune disease mainly resulting from the breakdown of self-tolerance. Bregs are critical in maintaining peripheral lymphocytes tolerance to auto-antigens. As such, we hypothesized that functional impairment of the Bregs mediates the pathogenesis of BP. To test this hypothesis, we detected compared the frequencies of Bregs in the peripheral blood from BP patients and from healthy controls, as well as investigated the immune functions of patient-derived Bregs *in vitro*. Unexpectedly, we found that the frequency of circulating CD19^+^CD24^hi^CD27^+^ Bregs was elevated in BP patients, compared with that in healthy controls. In addition, these Bregs displayed immunodeficiency in suppressing autoantibody production. Our findings provide novel insights into the pathogenesis of BP.

## Results

### Increased Breg frequency in BP patients

**Figure 1:**
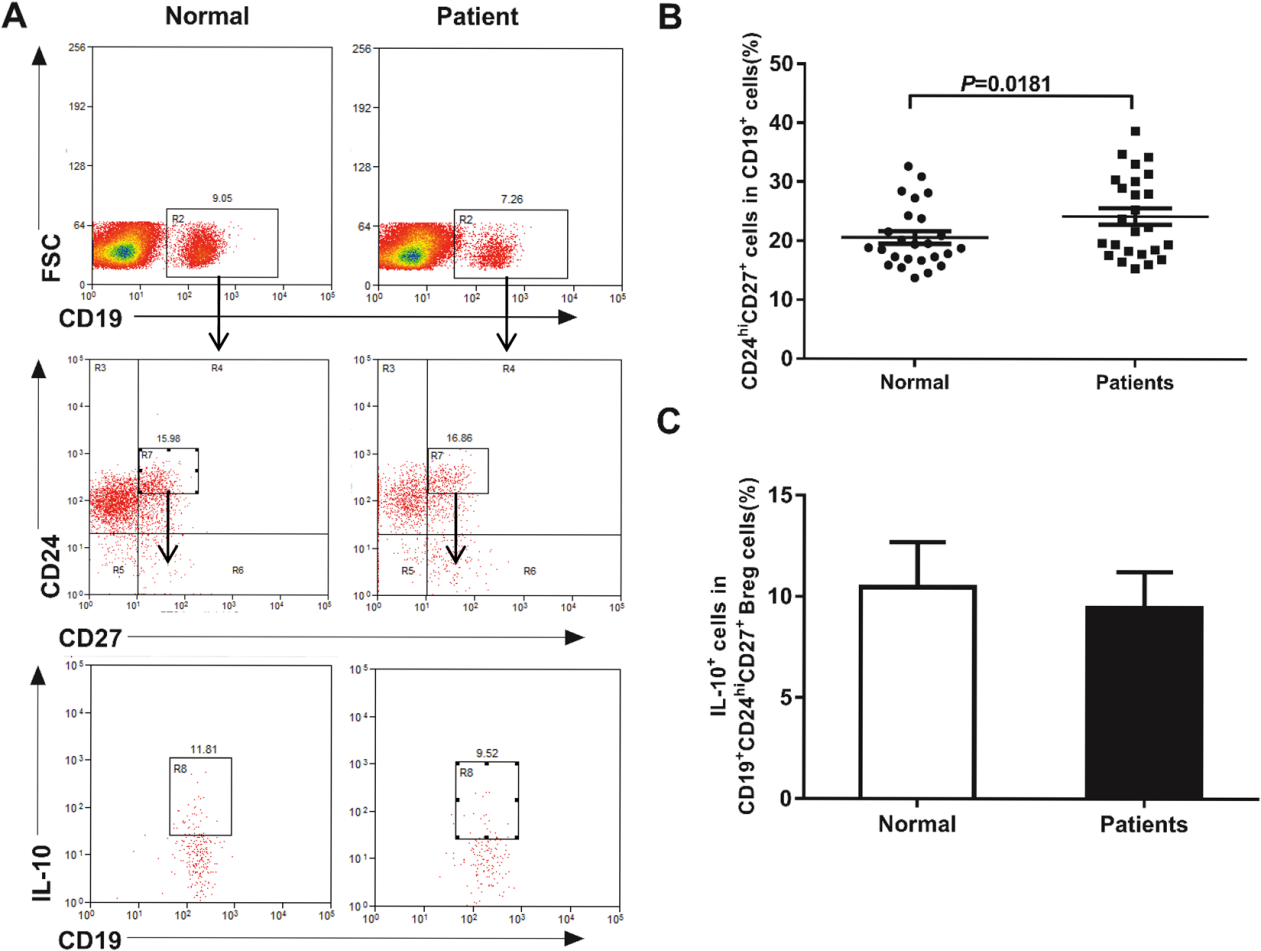
Frequency of Bregs in BP patients and healthy controls. (**A**) Representative FACS data of the frequency of CD19^+^CD27^+^ cells gated from lymphocytes in FSC/SSC dot plots (top), CD24^hi^ Bregs within gated CD19^+^CD27^+^ cells (middle) and IL-10^+^ Bregs within gated CD19^+^CD24^hi^CD27^+^ Bregs (bottom) of PBMCs frome BP patients and healthy controls. PerCP/Cy5.5 and PE mouse IgG1 isotype and FITC mouse IgG2a isotype were used as a negative control for immunofluorescence staining and flow cytometry assay. (**B**) Statistical analysis of the CD19^+^CD24^hi^CD27^+^/CD19^+^ cell ratios in indicated groups (n = 25 per group). (**C**) Statistical analysis of the IL-10^+^ cells within gated CD19^+^CD24^hi^CD27^+^ Bregs in indicated groups (n = 5 per group). *p* < 0.05 determined by two-tailed Student’s *t* test.

To observe the frequency of Bregs in BP patients, we identified Bregs as CD19^+^CD24^hi^CD27^+^ and IL-10^+^CD19^+^ by flow cytometry. We found that the frequency of CD19^+^CD24^hi^CD27^+^ Bregs (Fig. 1A and B) and IL-10^+^CD19^+^ Bregs (Sup Fig. 2A and B) were both significantly higher in BP patients compared with that in healthy controls (*p* < 0.05). To confirm the CD19^+^CD24^hi^CD27^+^ Bregs were actually IL-10-producing Bregs, we analyzed the expression of IL-10 in activated Bregs from BP patients and from healthy controls. The result showed that more than 10% of CD19^+^CD24^hi^CD27^+^ Bregs produced IL-10 (Fig. 1A and C), which was in line with the previous findings by Iwata *et al*.^10^. Our results indicated that the frequency of CD19^+^CD24^hi^CD27^+^ Bregs in BP patients was elevated compared with that in healthy controls.

### Modified function of Bregs in suppressing autoantibody production in BP patients

**Figure 2:**
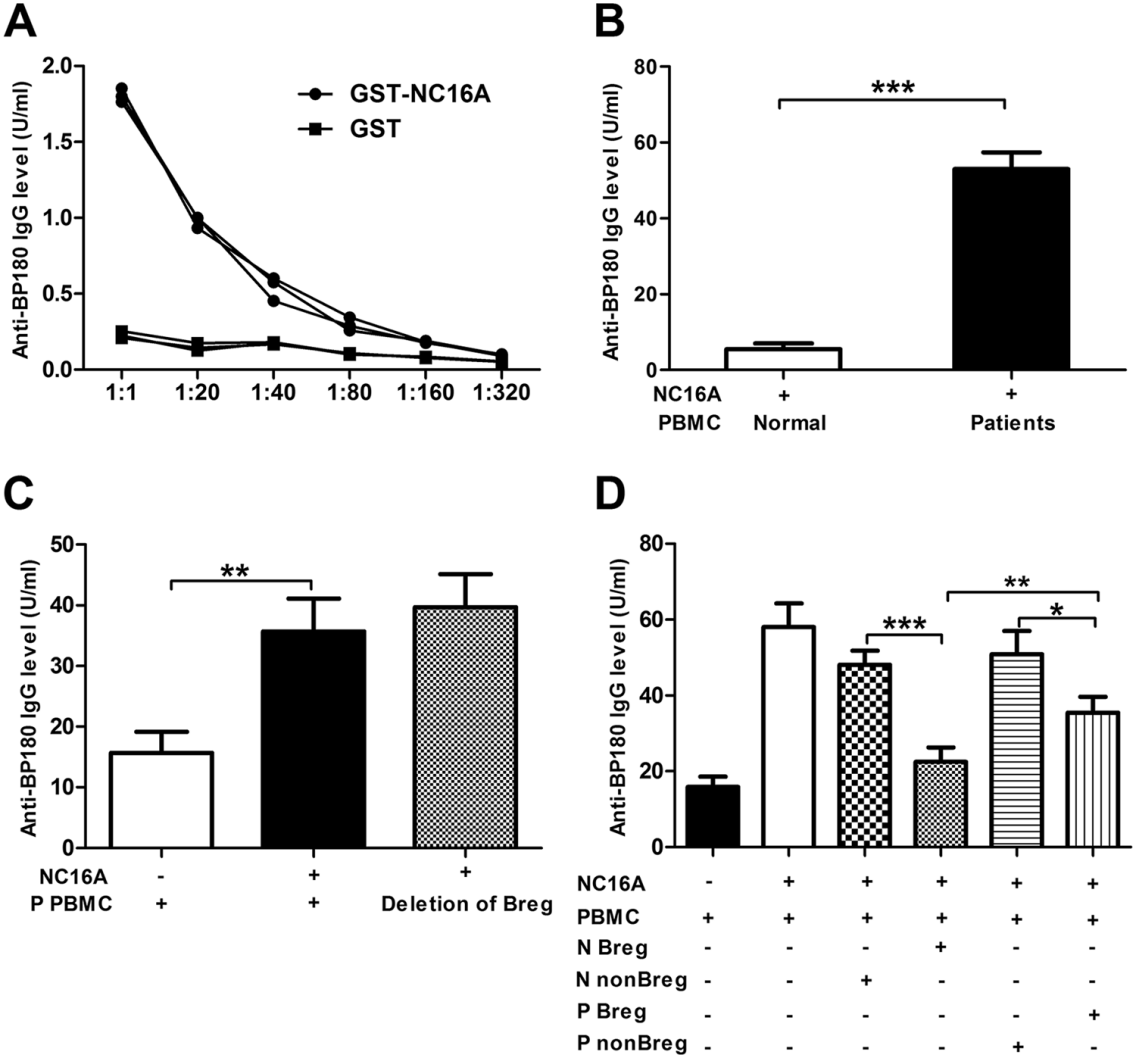
Suppressive function of Bregs. (**A**) ELISA analysis of the efficacy of purified NC16A binding to anti-BP180 antibodies. (**B**) PBMCs from healthy controls and BP patients were cultured with NC16A protein (5.0 μg/mL) for 72 h. ELISA analysis of the specific anti-NC16A antibody production (n = 5 per groups). (**C**) ELISA analysis of the specific anti-NC16A antibody production in PBMCs from BP patients with or without Breg deletion (n = 5). (**D**) PBMCs from BP patients were co-cultured with CD19^+^CD24^hi^CD27^+^ Bregs or CD19^+^CD24^−^CD27^−^ non-Bregs (3:1) from the third-part BP patients and healthy controls (n = 5 per groups). ELISA analysis of the specific anti-NC16A antibody production in the co-cultures. N stands for normal, P stands for patients. *p < 0.05, **p < 0.01 and ***p < 0.001 determined by paired version of two-tailed Student’s *t* test or one-way ANOVA followed by Bonferroni corrections for post hoc *t*-test.

To investigate the function of Bregs from BP patients in regulating immune responses, Bregs from BP patients and healthy controls were isolated and then observed for their effects on autoantibody production *in vitro*. In preparation for these assays, recombinant human BP180-NC16A proteins were expressed and purified, as shown in Sup Fig. 3. ELISA assays were performed to evaluate the binding activity of BP autoantibody to recombinant human BP180-NC16A. The results showed that GST-tagged NC16A bound patient autoantibodies robustly, whereas GST did not (Fig. 2A). Next, we incubated PBMCs from BP patients and from healthy controls respectively with the recombinant human BP180-NC16A protein. We found high levels of specific anti-BP180 antibody in cell culture supernatants of patient-derived PBMCs, whereas barely detectable anti-BP180 antibody titer in the cell culture supernatants of PBMCs from healthy controls (Fig. 2B).

To obverse the effect of Bregs on suppressing autoantibody production in BP patients, we compared anti-BP180 antibody titers in cell culture supernatants of the patient-derived PBMCs with or without the depletion of Bregs incubated with the recombinant human BP180-NC16A protein. Patient-derived BPMCs alone were used as negative controls. Notably, we observed no significant difference in the production of specific anti-BP180 antibody between patient-derived PBMCs with Bregs depletion and without Bregs depletion (Fig. 2C). These results suggested that Bregs from BP patients failed to suppress autoantibody production. To further determine the defective function of Bregs from BP patients, we co-cultured patient-derived PBMCs with Bregs from the BP patient or healthy controls, followed by incubation with BP180-NC16A. As expected, compared to Bregs from healthy controls, patient-derived Bregs showed impairment in suppressing autoantibody production (Fig. 2D). Our results suggest that the functional deficiency of Bregs may contribute to autoantibody production in BP.

### Modified function of BP Bregs to restrain CD4^+^ T cell activation

Activated CD4^+^ T cells are necessary to facilitate B cell proliferation and differentiation by producing pro-inflammatory cytokines, such as IL-4, IL-21, and TNF-α^17^. Previous studies showed that Bregs exhibit immunosuppressive function mainly by inhibiting CD4^+^ T cell proliferation and inflammatory cytokine production^18,19^. Given that we had found BP patient-derived Bregs have modified function in suppressing autoantibody production, we speculated that those Bregs have functional deficiency in restraining CD4^+^ T cell activation.

**Figure 3:**
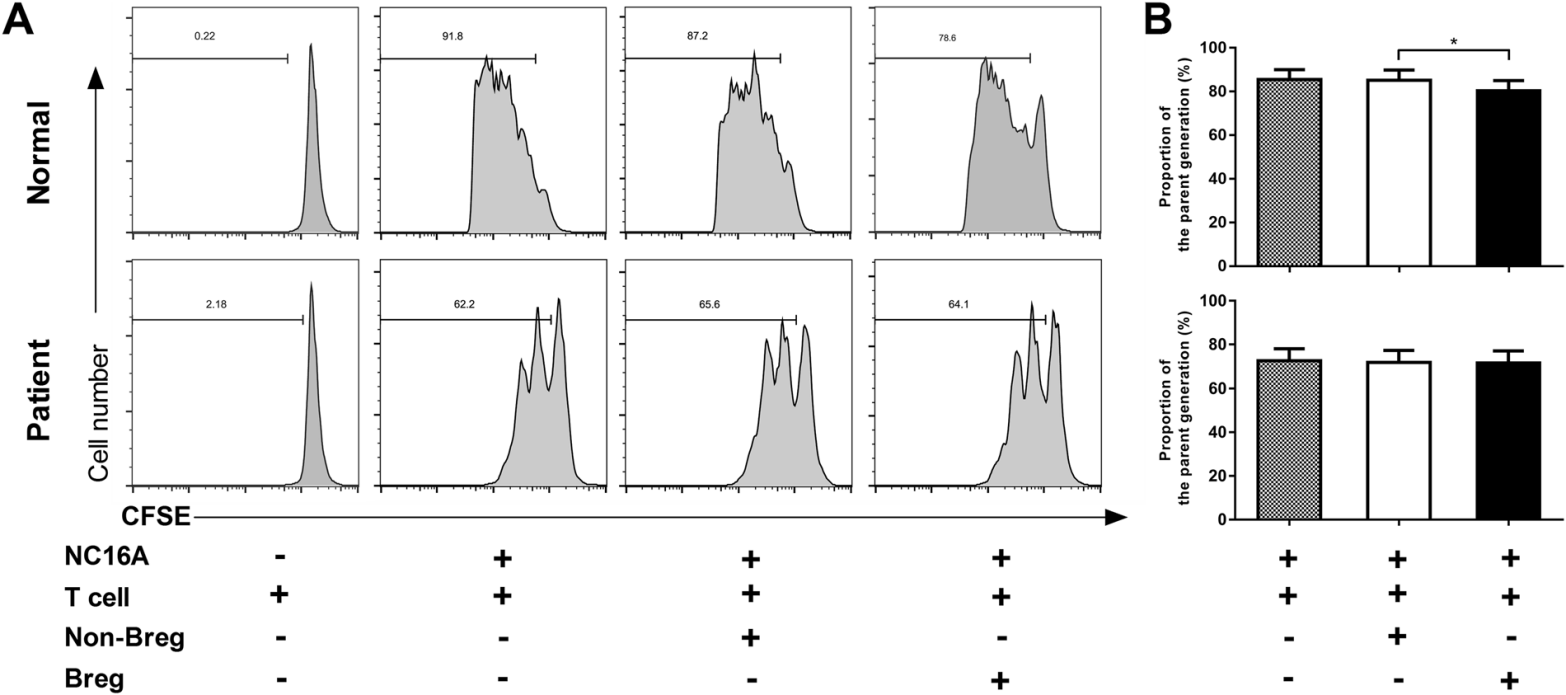
Effect of Bregs on the proliferation of T cells. CD4^+^ T cells labeled with CFSE were co-cultured with CD19^+^CD24^hi^CD27^+^ Bregs or CD19^+^CD24^−^CD27^−^ non-Bregs from BP patients and healthy controls. (**A**) Representative FACS data of the proliferation of CD4^+^ T cells based on CFSE signaling. (**B**) Statistical analysis of the dividing cells ratios of CD4^+^ T cells (n = 5 per groups). *p < 0.05 determined by paired version of one-way ANOVA followed by Bonferroni corrections for post hoc *t*-test.

To investigate the effect of the modified Bregs function on T cell proliferation in BP patients, CFSE labeled CD4^+^ T cells were co-cultured with Bregs and the proliferation rate of CD4^+^ T cells were determined by flow cytometry. We found that the proliferation rate of CD4^+^ T cells was decreased when co-cultured with Bregs from healthy controls, compared to the non-Breg groups. In contrast, the proliferation rate of CD4^+^ T cells did not differ when cultured with Bregs or non-Bregs from BP patients (Fig. 3A and B). These results indicate that healthy-derived Bregs was able to suppress CD4^+^ T cell proliferation, whereas BP patient-derived Bregs was lack of this function.

**Figure 4:**
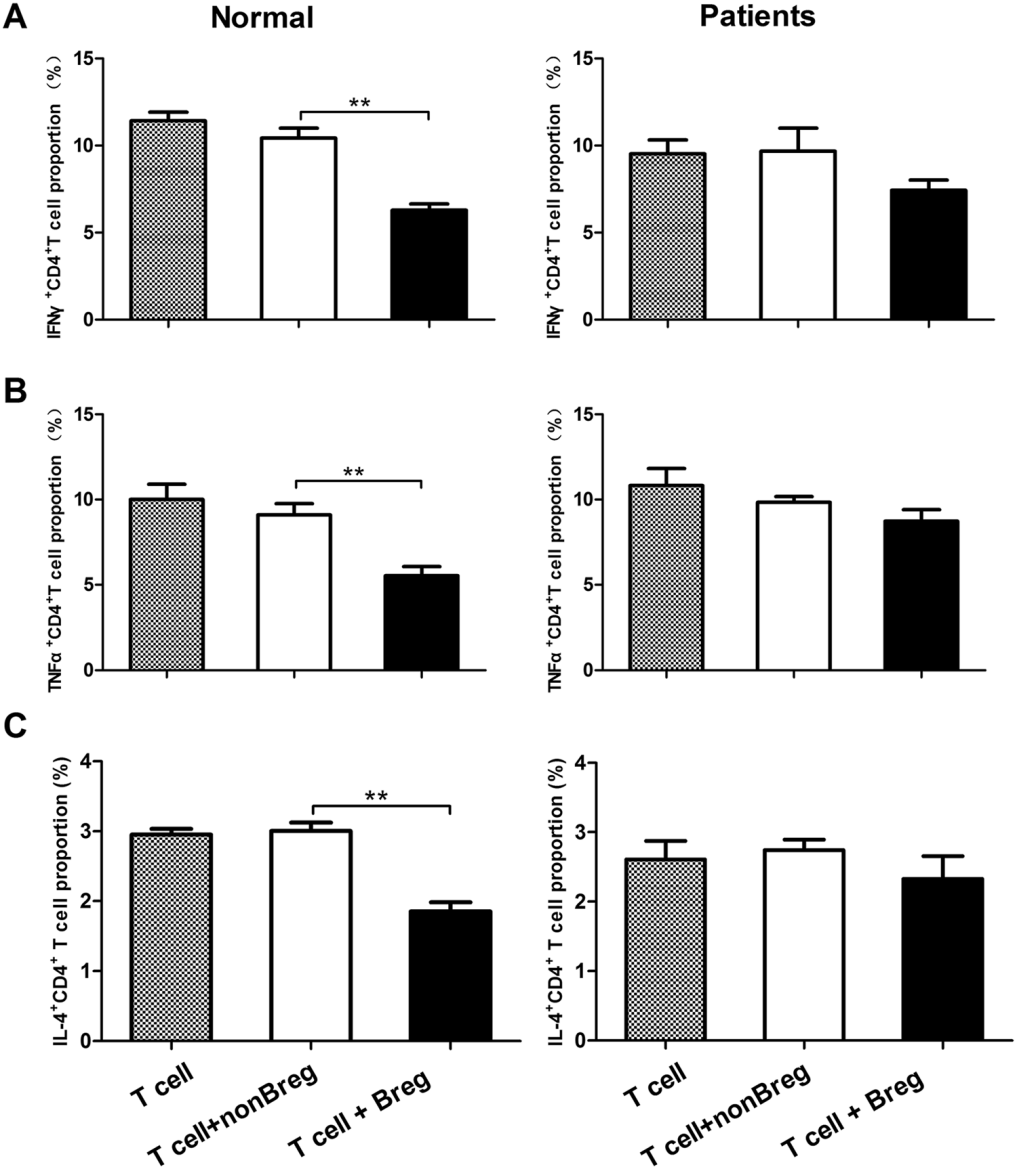
Effect of Bregs on the cytokine expression of T cells. CD4^+^ T cells co-cultured with CD19^+^CD24^hi^CD27^+^ Bregs or CD19^+^CD24^−^CD27^−^ non-Bregs from BP patients and healthy controls. Statistical analysis of the frequency of (**A**) CD4^+^IFN-γ^+^, (**B**) CD4^+^TNF-α^+^, and **(C)** CD4^+^IL-4^+^ (n = 5 per groups). **p < 0.01 determined by paired version of one-way ANOVA followed by Bonferroni corrections for post hoc *t*-test.

To further determine the effect of BP patient-derived Bregs on cytokine production of CD4^+^ T cells, purified CD4^+^ T cells were co-cultured with Bregs or non-Bregs and the expression of IFN-γ, TNF-α, and IL-4 in CD4^+^ T cells were assessed by flow cytometry. The resulted showed that CD4^+^ T cells produced significantly lower levels of these cytokines when co-cultured with Bregs than non-Bregs from healthy controls. In contrast, we observed no differences of these cytokines expressed by CD4^+^ T cells following the co-culture with either Bregs or non-Bregs from BP patients (Fig. 4 and Sup Fig. 4). In conclusion, these data indicate that healthy-derived Bregs suppress cytokine expression in CD4^+^ T cells, whereas patient-derived Bregs lack this function.

### TNF-α expression in Bregs from BP patient

**Figure 5:**
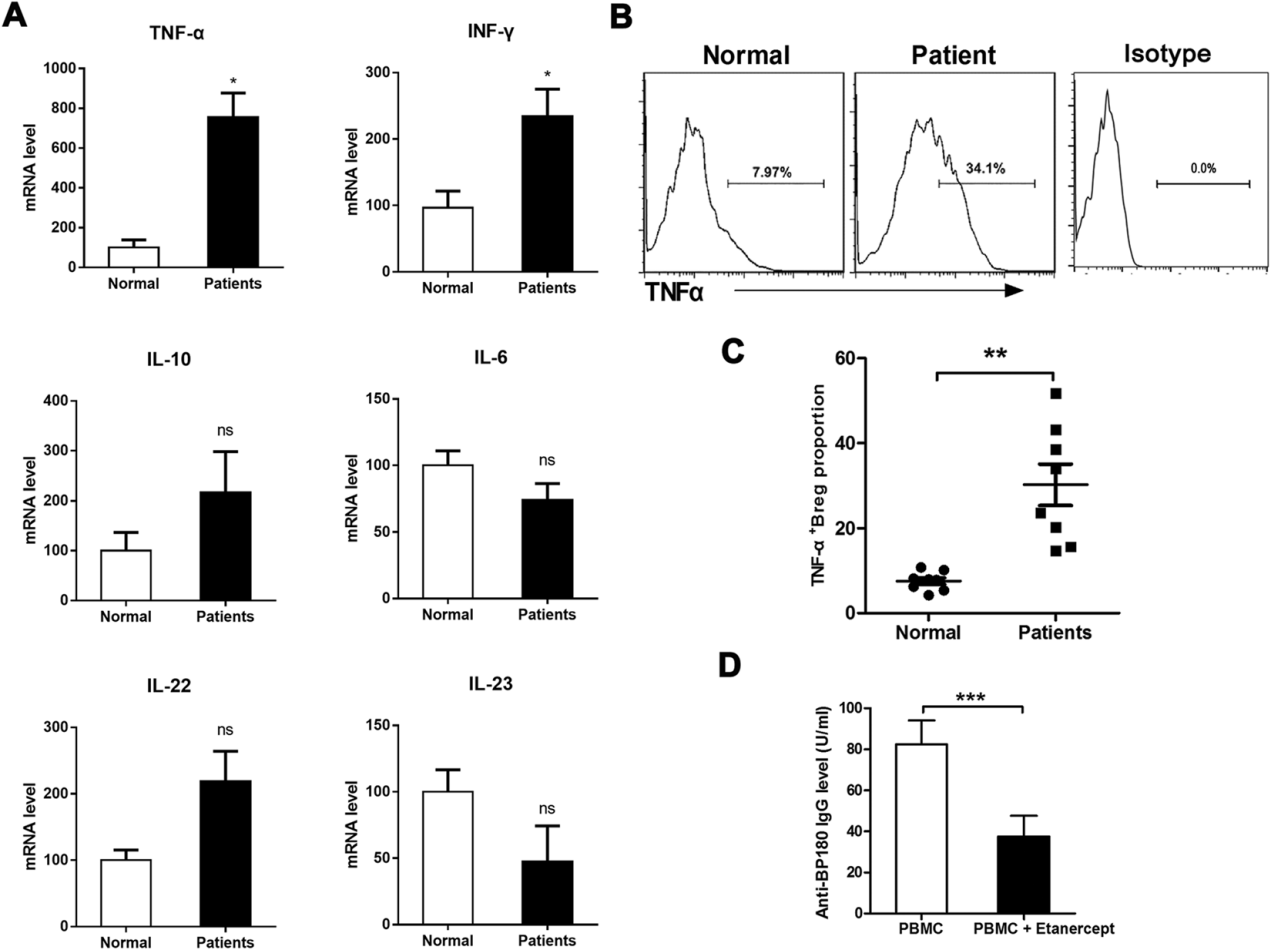
Modified function of Bregs were due to the expression of TNF-α. (**A**) Levels of TNF-α, IFN-γ, IL-10, IL-4, IL-22 and IL-23 mRNA in CD19^+^ CD24^hi^CD27^+^ Bregs after sorting from PBMCs of 5 patients and 4 healthy controls. (**B)** Representative FACS data of the frequency of TNF-α^+^ cells within gated CD19^+^CD24^hi^CD27^+^ Bregs from PBMCs of BP patients and healthy controls. APC/Cy7 Mouse IgG1 were used as a negative control for immunofluorescence staining and flow cytometry assay. (**C**) Statistical analysis of the frequency of TNF-α^+^ cells within gated CD19^+^CD24^hi^CD27^+^ Bregs in the indicated groups (n = 8 per groups). (**D**) Anti-BP180 antibody production in patient-derived PBMCs treated with etanercept. *p < 0.01, **p < 0.01 and ***p < 0.001 determined by two-tailed Student’s *t* test.

To investigate the underlying mechanism that responding to the dysfunction of Bregs in BP patients, we first observed the expression of inflammatory cytokines in the sorted CD19^+^CD24^hi^CD27^+^ Bregs from BP patients and from healthy controls. The real-time PCR results showed that TNF-α and IFN-γ were significantly up-regulated in the CD19^+^CD24^hi^CD27^+^ Bregs from patients compared with that in healthy controls (Fig. 5A). There were no significant difference of IL-10, IL-6, IL-22 and IL-23 mRNA between the two groups. In addition, we examined the expression of TNF-α in Bregs from BP patients and healthy controls by flow cytometry. The result showed that Bregs from patient produced high levels of TNF-α compared with that in healthy controls (Fig. 5B and C). Further, we added TNF-α antagonist etanercept into the co-culture system to observe its effect on the autoantibody production. The results showed that etanercept could inhibit the autoantibody production in the supernatant of patient-derived PBMCs (Fig. 5D). These results indicate that that Bregs from BP patient might express more inflammatory cytokines TNF-α and thus result to its modified function in suppressing autoantibody production.

## Discussion

In this study, we found that the frequency of circulating CD19^+^CD24^hi^CD27^+^ Bregs and IL-10^+^CD19^+^ Bregs were increased in BP patients. Moreover, our *in vitro* study suggested that Bregs from BP patient were defective in suppressing the CD4^+^ T cell activation and the specific autoantibody production. Furthermore, we found that these Bregs aberrantly produced high level of TNF-α in BP patients. Meantime, etanercept could down-regulate the BP autoantibody production. All these result highlight that Bregs in BP appear phenotypically pro-inflammatory by their cytokine profile and defective in immunosuppressive function, suggesting that Bregs play a pro-inflammatory role rather than a regulatory role in the pathogenesis of BP.

BP is a prevalent autoimmune blistering disease caused by autoantibodies against BP180. Studies have found that several subsets of immune cells, including Th1 cells, Th2 cells and Treg cells, are involved in the production of BP autoantibodies^20,21^. Our previous study also showed that the frequency of follicular T helper cells also contribute to BP by producing IL-21^22^. However, whether Breg cells are involved in the process is still unknown.

Bregs are a small population of B cells that participates in immunomodulation and in suppression of immune responses^23^. Directly, Bregs can interact with cognate T cell and control Treg cell induction^24^. Indirectly, Breg cells suppress the differentiation of Th1 and Th17 cells by suppressing pro-inflammatory cytokine production by dendritic cells^25^. In addition to expressing IL-10, Breg cells could express other immune-regulatory cytokines, such as TGF-β. Bregs derived TGF-β could induce both apoptosis of CD4^+^ and anergy in CD8^+^ in effector T cells^26^. Bosma A *et al*. reported that normalization of CD1d expression on newly repopulated CD19^+^CD24^hi^CD38^hi^ B cells corresponded to normalization of the invariant natural killer T (iNKT) cell number and function in SLE patients treated with rituximab, suggesting that Breg cells are critical in maintaining invariant natural killer (iNKT) cell homeostasis in humans^27,28^. Several mouse models of autoimmune diseases as rheumatoid arthritis or systemic lupus erythematosus (SLE) have confirmed the important role of Bregs in immunomodulations and in suppression of immune responses^26^.

To date, whether Breg cells uniquely derive from a specific progenitor or originate within conventional B cell subsets is still unclear, which make it difficult to identify the exact phenotypes of Bregs^29^. However, there is a consensus that Bregs suppress the immune response are mainly through IL-10 production or contact-dependent suppression manner^23^. In humans, both CD19^+^CD24^hi^CD38^hi^ and CD19^+^CD24^hi^CD27^+^ B cells are shown to control immune responses by secreting IL-10^10,30^. Nevertheless, these two subsets show distinct effects on T cells. Bregs with the immature B cell marker CD38 are considered to be able to induce the development of T regulatory cells while limiting the differentiation of Th1 and Th17 cells^31^. And Bregs with memory B cell marker CD27 are mainly responsible for suppressing the activity of CD4^+^ T cells. Previously, Iwata *et al*. confirmed that CD19^+^IL-10^+^ Bregs correspond to CD24^hi^CD27^+^ B cells in humans^10^. Hence, we focus on investigating the frequencies and immune-regulatory function of CD19^+^CD24^hi^CD27^+^ Bregs in BP patients.

Accumulated evidence has shown that the frequency of Bregs is aberrant in human autoimmune diseases^32^. Daien *et al*. found that the number of CD19^+^IL-10^+^ Bregs was decreased in patients with rheumatoid arthritis and inversely correlated with disease activity^33^. However, the frequency of CD24^hi^CD38^hi^ and CD24^hi^CD27^+^ Bregs was similar in patients with rheumatoid arthritis and healthy controls^34^. Thus, it is still controversial about the frequency of Bregs in autoimmune diseases. In our study, we focused on the change of Bregs in BP patients and found that both circulating CD19^+^CD24^hi^CD27^+^ Bregs and IL-10^+^CD19^+^ Bregs increased in BP patients compared to healthy controls. Further, we confirmed that CD19^+^CD24^hi^CD27^+^ Bregs from BP patients and healthy controls have the ability to secreting IL-10. Considering the regulatory function of Bregs on immune response, what is the role of elevated Bregs in the pathogenesis of BP? Our *in vitro* study provides evidence that CD19^+^CD24^hi^CD27^+^ Bregs from BP patients were defective in suppressing autoantibody production. This result were similar with the study in in patients with pemphigus that CD19^+^CD24^hi^CD38^hi^ Bregs were elevated in pemphigus patients and were defective regulatory function on T helper 1 cells^35^. Collectively, our study indicates that the modified function of Bregs may be a critical cause of BP.

Bregs are considered to suppress the activation of CD4^+^ T cells mainly by secreting IL-10^10^. In addition, IL-10 is an important anti-inflammatory cytokine and several studies showed that the level of IL-10 was decreased in T cell mediated autoimmune diseases, such as diabetes, psoriasis and rheumatoid arthritis, which indicates that decreased levels of IL-10 may cause activation of T cells, further leading to autoimmune diseases^36,37^. However, we found that CD19^+^CD24^hi^CD27^+^ Bregs produced comparable IL-10 between BP patients and healthy controls. Further, we showed that the number of IL-10 producing B cells were even increased in BP patients. Meanwhile, we noticed that the mRNA level of IL-10 was increased in PBMCs, while the serum level of IL-10 was comparable in BP patient compared with healthy controls (Sup Fig. 4c and d). It seems our results contradict with previous reports on T cell mediated autoimmune diseases. Nevertheless, several studies have shown that IL-10 level is elevated in some autoantibody-mediated autoimmune diseases, such as SLE and pemphigus vulgaris, and reduced IL-10 production is usually associated with remission^30,35^. In addition, IL-10 antagonists are effective in treating animal models of SLE^38^. All these suggest that modified function of Bregs that contribute to the pathogenesis of BP are independent on IL-10 production.

CD19^+^CD24^hi^CD27^+^ Bregs were mainly responsible for suppressing T cell activation, which is necessary for autoantibody production in B cells^10^. Thus, we investigated the effect of CD19^+^CD24^hi^CD27^+^ Bregs from patients on the activation of T cells. We found that BP patient-derived CD19^+^CD24^hi^CD27^+^ Bregs showed modified function in suppressing the proliferation and the pro-inflammatory cytokines secretion in CD4^+^ T cells. These findings suggest that Bregs contribute to the pathogenesis of BP through this shift in function to control the activation of CD4^+^ T cells.

Our result and other studies have indicated that Bregs showed modified function in immune-suppressive function in certain autoimmune disease^11^, but the underlining mechanism are still unclear. Recent study reported that pDCs induce the differentiation of Breg cells in an IFN-α-dependent manner in health individuals and this kind of regulation are defective in SLE patients which leading to impairment of Bregs in immune-suppressive function^39^. However, our result showed that Bregs gain the function to produce more inflammatory cytokines (mainly TNF-α, INF-γ) in BP patients. It seems controversial between the pro-inflammatory phenotype and weak immune-suppressive function of Breg in BP patients. However, recent studies proposed that immunosuppression is not the purview of a devoted Breg cell lineage with a specific phenotype but rather is the outcome of the dynamic balance between multiple B cell subsets and other cells of the immune system^26^. Functional flexibility of Breg subsets maybe responsible for the contradiction between phenotype and function in our study. In addition, more resolution of Breg phenotype will be helpful to understand the function in BP pathogenesis.

TNF-α is considered to be a critical cytokine for the initiation and perpetuation of inflammation. Studies showed that TNF-α contributes to many human autoimmune diseases by promoting the expansion and survival of T cells, including diabetes, rheumatoid arthritis, and psoriasis^13^. In healthy controls, TNF-α is mainly produced by pro-inflammatory lymphocytes and β-glucan-activated B lymphocytes could up-regulate pro-inflammatory cytokines TNF-α^14^. we further found that TNF-α blockade could suppress the autoantibody production, suggesting that defective function of Bregs is due to the production of TNF-α. Therefore, we speculated that Bregs play a pro-inflammatory role rather than a regulatory role in the pathogenesis of BP^40^.

However, there have several limitations. Firstly, as the minimal difference of increased frequency in Bregs, it just reflect a trend to an increased proportion of one particular type of Bregs in BP. The mechanistic and functional importance of this trend still need further investigation. Secondly, this study illustrated the pro-inflammatory function of Bregs and the crucial role of TNF-α in Bregs dysfunction in BP. However, it is unclear whether these findings are a general mechanisms of autoimmunity or just a phenomena of BP. Current finding would be more significance by including other antibody mediated autoimmune patients in this study. Finally, current result only uncovered the change of TNF-α in dysfunctional Bregs in BP. It would be more valuable to discuss the molecules or pathways changes with high throughput approach.

In summary, we found that frequency of circulating CD19^+^CD24^hi^CD27^+^ Bregs were increased in BP patients, and these cells were defective in suppressing autoantibody production and CD4^+^ T cell activation. Moreover, we provide strong evidence to suggest that the decreased suppressive capacity of Bregs is due to aberrant TNF-α production, rather than decreased IL-10 production. Our results provide new insights into the role of Bregs in the pathogenesis of BP.

## Methods

### Patients and healthy controls

Blood and serum samples were obtained from 41 newly diagnosed BP patients (age range: 41–84, 17 males and 24 females). These patients had not yet received steroids or other immunosuppressive therapies. Samples from 34 healthy volunteers were included in parallel as controls group (age range: 36–75, 18 males and 16 females). All the patients and healthy volunteers involved have signed an informed consent prior to initiation of the study. All experiments were performed in accordance with the relevant guidelines and regulations approved by the Institutional Ethics and Research Advisory Committee, the Fourth Military Medical University, Xi’an, China.

### Human cell isolation and cell culture

Peripheral blood mononuclear cells (PBMCs) were isolated by using lymphoprep (Dakewe, Shenzhen, CHN) according to the manufacturer’s protocols. B cells and CD4^+^ T cells (> 85% purity) were isolated by using magnetic beads (BD, NJ, USA). Bregs (CD19^+^CD24^hi^CD27^+^), non-Bregs (CD19^+^CD24^−^CD27^−^) and PBMCs without Bregs were isolated by fluorescence-activated cell sorting (FACS Calibur; BD) using monoclonal anti-human CD19-PerCP/Cy5.5 (clone: SJ25C1), CD24-FITC (clone: ML5) and CD27-PE (clone: M-T271) antibodies (BioLegend, CA, USA) (Sup Fig. 1). PerCP/Cy5.5 and PE mouse IgG1 isotype and FITC mouse IgG2a isotype control were used for immunofluorescence staining and flow cytometry assay. Isolated cells were then cultured in RPMI 1640 supplemented with 10% fetal calf serum (FCS, Gibco, CA, USA).

### Flow cytometric analysis of Bregs

Fresh PBMCs were incubated with anti-human CD19-PerCP/Cy5.5, CD24-FITC, CD27-PE antibodies for 30 min at 4 °C, then fixed with Fixation Solution (4 A Biotech Co., Ltd, Beijing, China) for 30 min, and analyzed by flow cytometry (FACS Calibur, BD). To investigate IL-10 expression in Bregs, fresh PBMCs were incubated with LPS (10 μg/mL; Sigma, MO, USA), CD40L (1 μg/mL; R&D Systems, MN, USA) for 48 hours to stimulate cell activation, then with PMA (50 ng/mL; Sigma), ionomycin (1 μg/mL; Sigma) and Golgi Stop™ (0.2 μL; BD) for 4 hours. After labeled with anti-human CD19-PerCP/Cy5.5, CD24-FITC, CD27-PE antibodies, PBMCs were fixed and permeabilized using the Fixation/Permeabilization solution (eBioscience) and Permeabilization Buffer (eBioscience) according to the manufacturer’s instructions. Finally, they were stained with IL-10-APC antibody (BioLegend) for 30 min at 4 °C, and analyzed by flow cytometry (FACS Calibur, BD).

### NC16A protein expression and purification

The GST-NC16A fusion protein was expressed in the prokaryotic expression vector, pGEX-4T-BP180NC16A. GST purification modules were used to purify bacterial lysates and elute protein from beads (GE Healthcare, Shanghai, CHN). Proteins concentration were measured by ultrafiltration through Amicon Ultra 15-mL filters. The molecular weight and purity of the purified GST-NC16A fusion protein were estimated by SDS-PAGE electrophoresis. Proteins (10 μg) were separated by SDS-PAGE and stained by coomassie brilliant blue solution. Proteins were detected using a chemiluminescence detection kit (KPL, Gaithersburg, MD). Serial dilutions of patient-derived serum samples were added to a 24-well plate pre-coated with the GST-NC16A fusion protein. The affinity of BP180-NC16A binding to autoantibodies were analyzed by enzyme-linked immune sorbent assay (ELISA).

### Function of Bregs in suppressing autoantibody production

To identify anti-NC16A antibody specifically produced by BP patient-derived PBMCs, PBMCs from BP patients and healthy controls were cultured in 24-well plates (1.5 × 10^6^ cells per well) pre-coated with purified NC16A protein (5.0 μg/mL, 300 μL per well). Next, to test the effect of Bregs on autoantibody production of BP patients, PBMCs isolated from BP patients with or without Bregs depletion were cultured in 24-well plates (1.5 × 10^6^ cells per well) pre-coated with purified NC16A protein (5.0 μg/mL, 300 μL per well). In addition, patient-derived PBMCs (1 × 10^5^ per well) were co-cultured with Bregs (3 × 10^4^ cells per well) from healthy controls or another patients in 96-well plates coated with the NC16A protein (5.0 μg/mL, 100 μL per well). Then, the supernatants were harvested 72 h later, and specific anti-NC16A antibody levels were determined by ELISA assay.

### Function of Bregs in suppressing T cell activation

To investigate the effect of Bregs on suppressing T cell activity, CD4^+^ T cells (1 × 10^7^) were first stained with CFSE (1.5 μM) for 8 minutes at room temperature. Then an equal volume of pre-warmed FBS were added and incubated in 37 °C for 10 min for efflux. CFSE stained T cells were washed for two times with 2% FBS after centrifuge. CD4^+^ T cells (5 × 10^4^ per well) labeled with CFSE were co-cultured with Bregs or nonBregs (5 × 10^4^ per well) from BP patients or healthy controls in 96-well U-bottom plates. These plates were coated with anti-CD3 (10 μg/mL, 100 μL per well; Catalog Number: 14–0038, Clone: UCHT1; eBioscience). Then recombinant IL-2 (100 units; R&D Systems) and anti-CD28 antibodies (1 μg/mL; eBioscience) were added to stimulate T cell activation for 72 hours. The proliferation of CD4^+^ T cells was analyzed by flow cytometry assays based on the CFSE signal. To investigate the cytokines expression in CD4^+^ T cells, PMA (50 ng/mL; Sigma), ionomycin (1 μg/mL; Sigma) and Golgi Stop™ (0.2 μL; BD) were added into the culture system for 4 hours. Then cells were labeled by using anti-human CD4-PE/Cy7 (clone: OKT4), IFN-γ-APC (clone: 4 S.B3), IL-4-PE (clone: 8D4-8) and TNF-α-APC/Cy7 (clone: MAb11) antibodies (BioLegend), and analyzed by flow cytometry (FACS Calibur, BD).

### Flow cytometric analysis of cytokines expression in T cells, Bregs and etanercept treatment

PBMCs were incubated with PMA (50 ng/mL; Sigma), ionomycin (1 μg/mL; Sigma) and Golgi Stop™ (0.2 μL; BD) for 4 hours. Then they were labeled with anti-human CD19-PerCP/Cy5.5, CD24-FITC, CD27-PE, TNF-α-APC/Cy7 antibodies (BioLegend), and analyzed by flow cytometry (FACS Calibur, BD).

To investigate the role of TNF-α in autoantibody production, patient-derived PBMCs were treated with etanercept (Pfizer, USA) to block the effect of TNF-α. Because the therapeutic range of residual serum concentration is between 1 and 10 μg/ml, we used 10 μg/ml of etanercept to incubate PBMCs for 72 hours. Then the supernatants were collected, the autoantibody production were determined by Elisa assay.

### Enzyme-linked immune sorbent assay (ELISA) and quantitative PCR (qPCR)

Levels of IL-10 in serum or culture supernatants were measured by using a human IL-10 ELISA kit according to the manufacturer’s protocol (R&D Systems). The levels of anti-NC16A antibody were measured using a human ELISA kit (Medical&Biological laboratories, LTD. KDX Nagoya Sakae Bldg, Japan). The optical density (OD) was read at 450 nm in a microplate reader (Bio-Rad Model 680, CA, USA).

Total RNA from the PBMCs and sorted Bregs was extracted by using TRIzol (Takara) according to the manufacturer’s instruction. cDNAs were then generated using a PrimeScript RT regent kit with 1 μg of total RNA per reaction. Quantitative real-time PCR was conducted using the SYBR Premix Ex Taq. β-Actin was used as an internal control. Samples were normalized to the independent control housekeeping gene β-actin and were reported according to the ΔΔCT method as RNA fold increase: 2^ΔΔCT^ = 2^ΔCT sample^ − 2^ΔCT reference^. The sequences of each primers are given in Supplementary Table S2.

### Statistical analysis

Aggregate data are presented as the mean ± SD. The two-tailed Student’s *t* test was used to analyze difference between two groups, and more than three groups were analyzed by one-way ANOVA followed by Bonferroni corrections for post hoc *t*-test. All analyses were performed using GraphPad Prism software, version 5 (GraphPad Software Inc, CA, USA).

## Electronic supplementary material

Supplementary Dataset 1
