# Supplementary material for: Dysfunction of CD19+CD24hiCD27+ B regulatory cells in patients with bullous pemphigoid
Source: Sci Rep. 2018 Jan 15;8:703. doi: 10.1038/s41598-018-19226-z (PMC5768798; doi:10.1038/s41598-018-19226-z)
Supplement: Supplementary file 1 — Supplementary Dataset 1 [file 41598_2018_19226_MOESM1_ESM.doc]

**Dysfunction of CD19^+^CD24^hi^CD27^+^ B regulatory cells in patients with bullous pemphigoid**

Zhenfeng Liu^1,2^, Erle Dang^1,2^, Bing Li^1,2^, Hongjiang Qiao^1^, Liang Jin^1^, Jieyu Zhang^1^, Gang Wang^1^,*

^1^ Department of Dermatology, Xijing Hospital, Fourth Military Medical University, 127 Changlexi Road, Xi’an 710032, China

^2^ These authors contributed equally to this work.

*Corresponding author: Department of Dermatology, Xijing Hospital, Fourth Military Medical University, Xi'an, Shaanxi Province, China. Tel. & fax: +86-29-84775401. E-mail address: xjwgang@fmmu.edu.cn (Gang Wang)

**
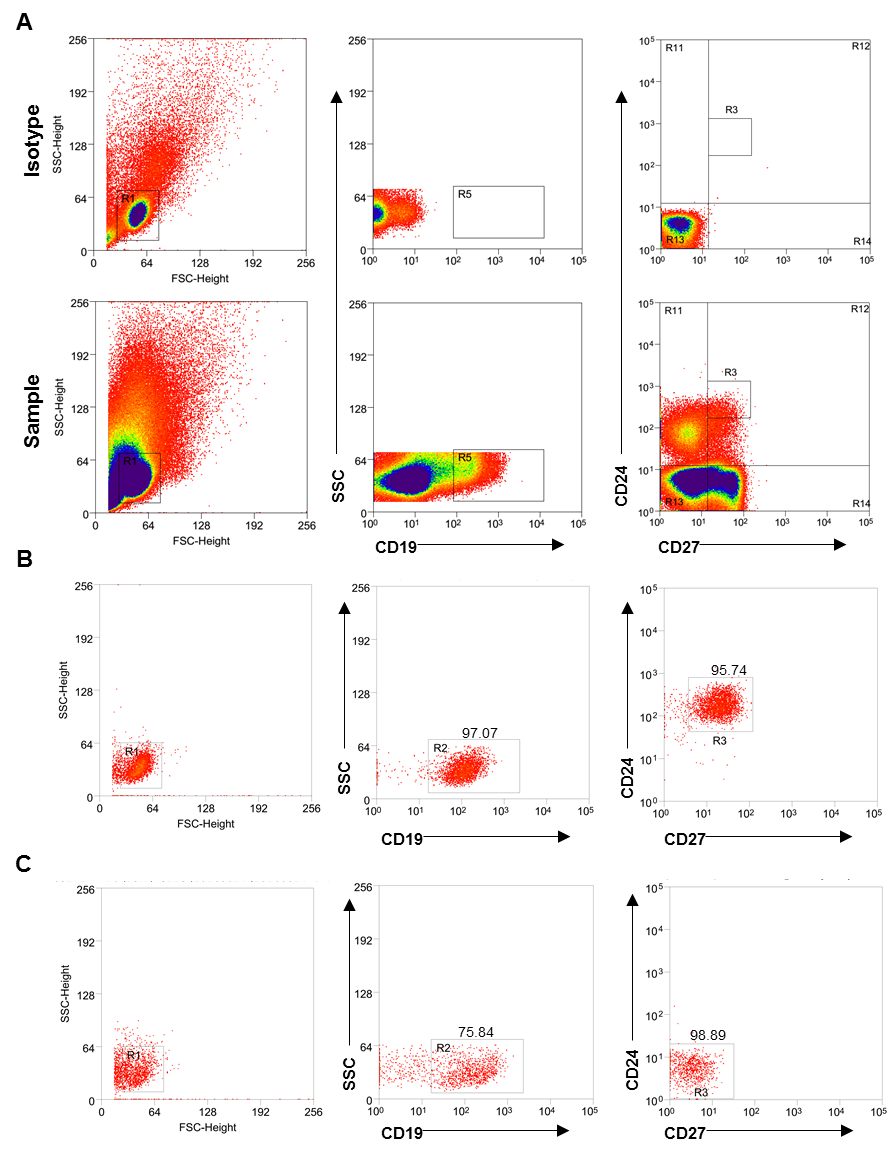
**

**Sup Fig 1. Purity of sorted CD19^+^CD24^hi^ghCD27^+^ and CD19^+^CD24^-^CD27^-^ cells. (**A) PBMCs from BP patients or healthy controls were labeled with anti-CD19 anti-CD24 and anti-CD27 fluorescent antibody, and the CD19^+^CD24^hi^ghCD27^+^ Bregs (Gate R3) and CD19^+^CD24^-^CD27^-^ cells (Gate R13) were sorted by flow cytometry. (B)(C) The purity of these two group of cells were analyzed by flow cytometry. Representative FACS data of the purity of CD24^hi^ghCD27^+^ and CD24^-^CD27^-^ in CD19^+^ B cells.


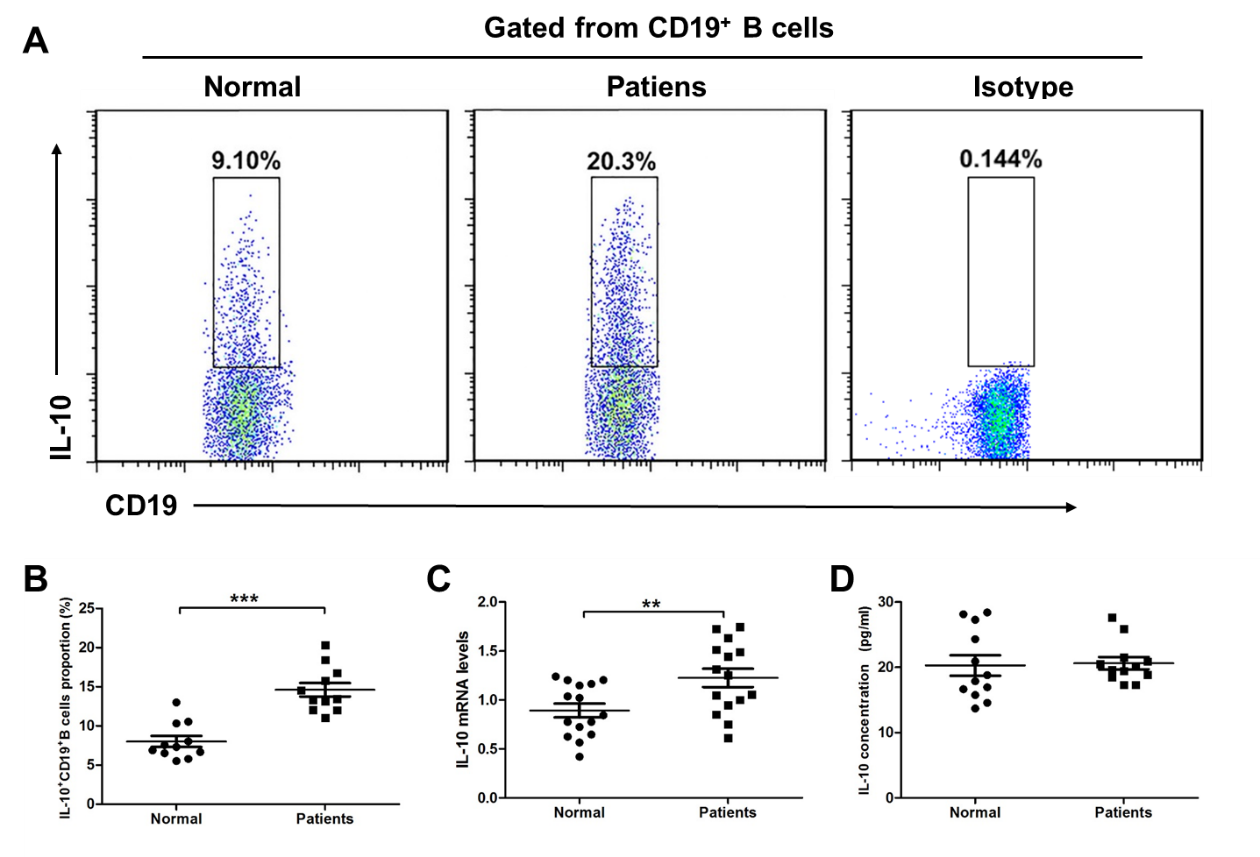
 **Sup Fig 2. CD19^+^IL-10^+^ B cells and IL-10 production in BP patients and healthy controls.** (A)Representative FACS data of the frequency of CD19^+^IL-10^+^ B cells in PBMCs from BP patients and healthy controls. (B) Statistical analysis of the CD19^+^IL-10^+^ /CD19^+^ cell ratios in the indicated groups (n = 11 per groups). (C) The mRNA level of IL-10 in PBMCs from BP patients and healthy controls (n =15 per groups). (D) The serum level of IL-10 in BP patients and healthy controls (n = 12 per groups). ***p* < 0.01 and ****p* < 0.001 determined by two-tailed Student’s *t* test.


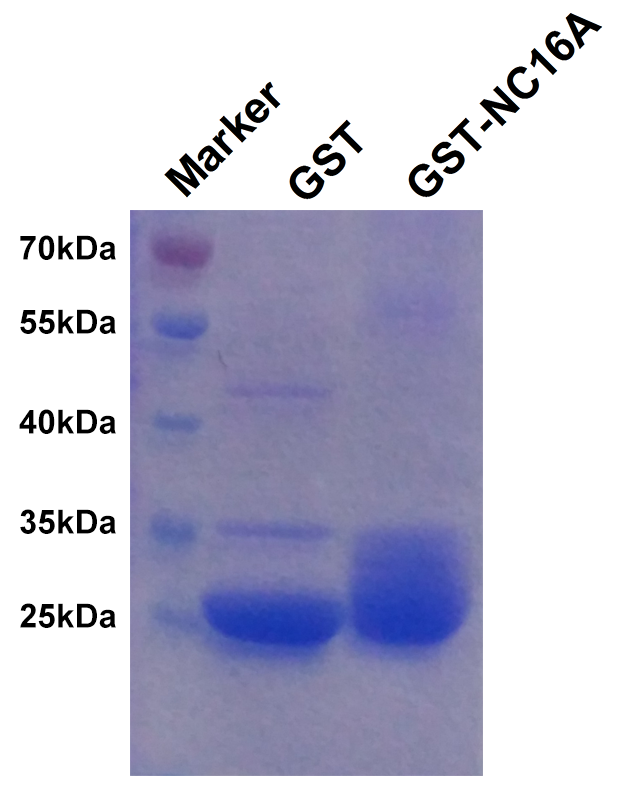


**Sup Fig 3. SDS-PAGE electrophoresis and coomassie brilliant blue staining of purified GST-NC16A fusion protein.**

**
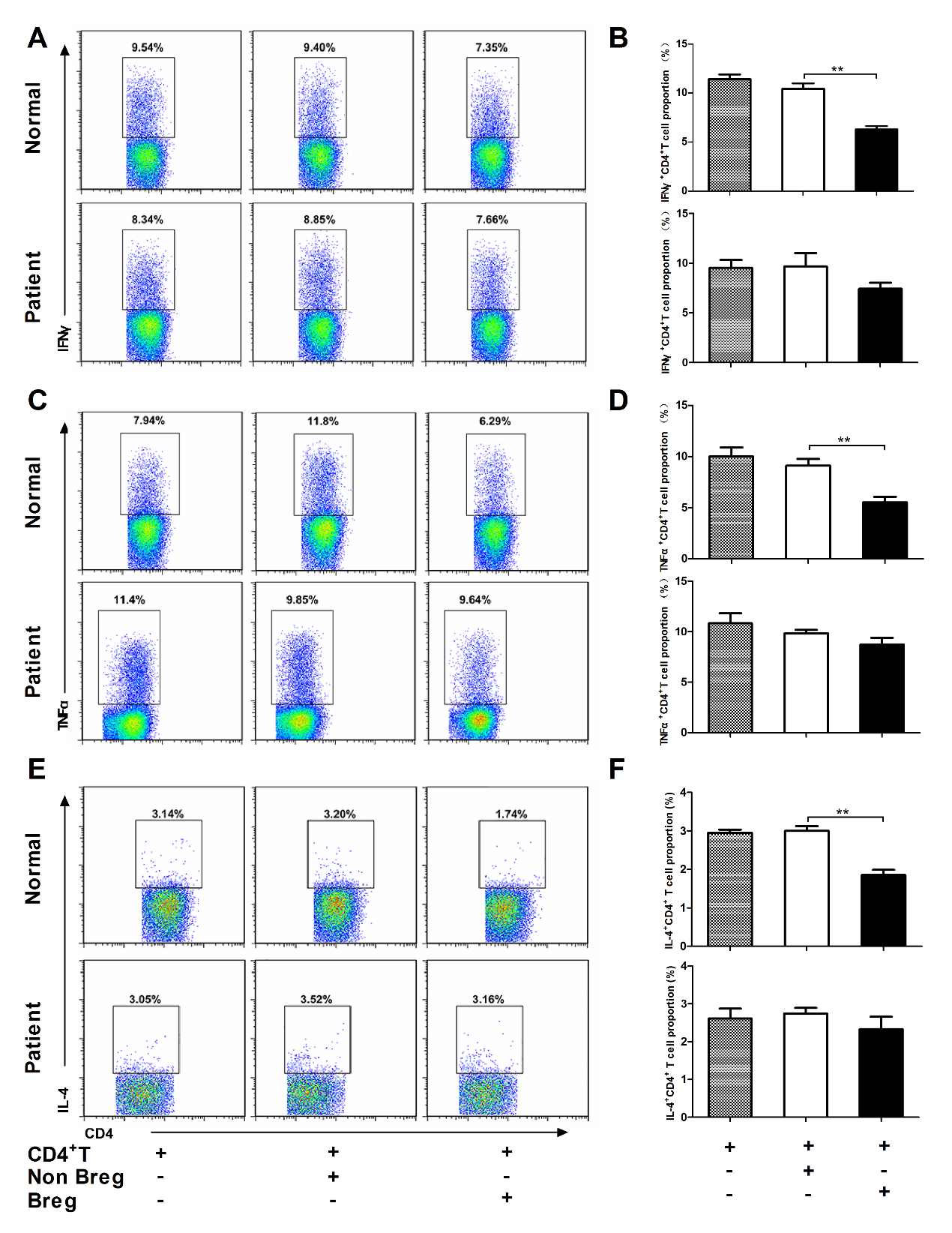
**

**Sup Fig 4. Effect of Bregs on the cytokine production of T cells.**

CD4^+^ T cells were co-cultured with CD19^+^CD24^hi^CD27^+^ Bregs or CD19^+^CD24^-^CD27^-^ non-Bregs from BP patients and healthy controls. Cells were harvested and stain with anti-human CD4-PE/Cy7, IFN-γ-APC, IL-4-PE and TNF-α-APC/Cy7 antibodies. Representative FACS data of the frequency of A) IFN-γ^+^, C) TNF-α^+^, and E) IL-4^+^ cells from CD4^+^ T cells. Statistical analysis of the frequency of B) CD4^+^IFN-γ^+^, D) CD4^+^TNF-α^+^, and F) CD4^+^IL-4^+^ (n = 5 per groups). ***p* < 0.01 determined by one-way ANOVA followed by Bonferroni corrections for post hoc *t*-test.

**S1 Table 1. Information of Patients with BP**

|  | Sex | Age | Anti-BP180 antibody (U/ml) |
| --- | --- | --- | --- |
| 1 | Female | 82 | 56 |
| 2 | Female | 54 | 66 |
| 3 | Female | 58 | 86 |
| 4 | Male | 47 | 87 |
| 5 | Male | 48 | 50 |
| 6 | Male | 76 | 6 |
| 7 | Female | 34 | 21 |
| 8 | Female | 35 | 25 |
| 9 | Female | 58 | 23 |
| 10 | Female | 64 | 67 |
| 11 | Male | 70 | 167 |
| 12 | Male | 66 | 218 |
| 13 | Female | 81 | 84 |
| 14 | Male | 68 | 161 |
| 15 | Male | 61 | 60 |
| 16 | Male | 67 | 38 |
| 17 | Male | 84 | 2 |
| 18 | Female | 56 | 21 |
| 19 | Female | 71 | 105 |
| 20 | Female | 72 | 61 |
|  | Sex | Age | Anti-BP180 antibody (U/ml) |
| 21 | Female | 73 | 61 |
| 22 | Female | 75 | 127 |
| 23 | Male | 82 | 60 |
| 24 | Female | 72 | 167 |
| 25 | Male | 79 | 210 |
| 26 | Male | 64 | 122 |
| 27 | Male | 65 | 132 |
| 28 | Female | 45 | 83 |
| 29 | Female | 45 | 19 |
| 30 | Female | 41 | 20 |
| 31 | Female | 41 | 12 |
| 32 | Male | 53 | 108 |
| 33 | Male | 54 | 49 |
| 34 | Female | 48 | 214 |
| 35 | Female | 44 | 198 |
| 36 | Female | 47 | 15 |
| 37 | Male | 61 | 52 |
| 38 | Female | 73 | 97 |
| 39 | Female | 57 | 103 |
| 40 | Male | 45 | 48 |
| 41 | Female | 59 | 70 |

**S1 Table 2. Primers and Sequences Used in the Experiment**

| **Primers for Real Time PCR** | |
| --- | --- |
| TNF-a-F | TCTCCCCTGGAAAGGACAC |
| TNF-a-R | AAGAGGCTGAGGAACAAGCA |
| IL-10-F | CTAACCTCATTCCCCAACCA |
| IL-10-R | CTCAGCCTCCCAAAGTGCT |
| IFN-r-F | GAGTGTGGAGACCATCAAGGA |
| IFN-r-R | GTATTGCTTTGCGTTGGACA |
| IL23-F | GCCAGCAGCTTTCACAGAAG |
| IL23-R | CCAGTAGGGAGGCATGAAGC |
| IL-6-F | TTCGGTCCAGTTGCCTTCT |
| IL-6-R | GGTGAGTGGCTGTCTGTGTG |
| IL-22-F | TTGGAGAACTGGATTTGCTGT |
| IL-22-R | ATCGCTTTGGGGCATCTAA |
| Actin-F | GGCTACAGCTTCACCACCAC |
| Actin-R | TGCGCTCAGGAGGAGC |
